# A multicenter prospective randomized study comparing the incidence of periprocedural cerebral embolisms caused by catheter ablation of atrial fibrillation between cryoballoon and radiofrequency ablation (Embo‐Abl study): Study design

**DOI:** 10.1002/clc.23949

**Published:** 2022-11-22

**Authors:** Koji Miyamoto, Koshiro Kanaoka, Yasutoshi Ohta, Masue Yoh, Hiroki Takahashi, Rena Tonegawa‐Kuji, Yuichiro Miyazaki, Keiko Shimamoto, Akinori Wakamiya, Nobuhiko Ueda, Kenzaburo Nakajima, Tsukasa Kamakura, Mitsuru Wada, Kohei Ishibashi, Yuko Inoue, Satoshi Nagase, Takeshi Aiba, Akihisa Narai, Tomohiro Nakase, Masatoshi Koga, Tetsuya Fukuda, Naoya Kataoka, Masahiko Takagi, Kengo Kusano

**Affiliations:** ^1^ Department of Cardiovascular Medicine National Cerebral and Cardiovascular Center Suita Japan; ^2^ Center for Cerebral and Cardiovascular Disease Information, Open Innovation Center National Cerebral and Cardiovascular Center Suita Japan; ^3^ Department of Radiology National Cerebral and Cardiovascular Center Suita Japan; ^4^ Department of Medicine II Kansai Medical University Moriguchi Japan; ^5^ New Development Group Hashimoto Electronic Industry Co., Ltd Matsusaka Japan; ^6^ Department of Cerebrovascular Medicine National Cerebral and Cardiovascular Center Suita Japan; ^7^ Second Department of Internal Medicine University of Toyama Toyama Japan

**Keywords:** atrial fibrillation, cerebral embolism, cryoballoon ablation, radiofrequency ablation

## Abstract

**Background:**

Although catheter ablation (CA) has become a standard therapeutic approach to atrial fibrillation (AF), it imposes a low but relevant risk of thromboembolic complications of around 0.5%–1%, including ischemic strokes, and has an additional risk of clinically silent cerebral embolisms (SCEs) of 10%–40%. Both cryoballoon (CB) and radiofrequency (RF) ablation are routinely used clinically worldwide, yet there are few prospective data comparing the incidence of cerebral embolism after CA of AF between CB and RF ablation.

**Methods:**

The aim of the Embo‐Abl study will be to compare the incidence of cerebral embolisms on 3 T diffusion‐weighted image magnetic resonance imaging (MRI) after CA of AF between CB and RF ablation in patients with AF in a prospective, multicenter, open‐label, controlled, randomized fashion. The primary endpoint of the Embo‐Abl study will be the occurrence of MRI‐detected SCE 1–3 days after CA. The patients will be registered and randomly assigned to either the CB or RF ablation group in a 1:1 ratio. The study cohort will include 230 patients with AF from a multicenter in Japan.

**Results:**

The results of this study are currently under investigation.

**Conclusion:**

The Embo‐Abl study will be the first to compare the incidence of periprocedural cerebral embolisms caused by CA of AF between CB and RF ablation in a prospective, multicenter, randomized, controlled fashion.

**Abbreviations**
ACT: activated clotting time
ADC: apparent diffusion coefficient
AE: adverse event
AF: atrial fibrillation
AT: atrial tachyarrhythmia
BP: blanking period
CA: catheter ablation
CB: cryoballoon
CRB: Certified Review Board
CRF: Case Report Form
DOAC: direct oral anticoagulants
DSMB: Data and Safety Monitoring Board
DWI: diffusion‐weighted image
FLAIR: fluid‐attenuated inversion recovery
jRCT: Japan Registry of Clinical Trials
LA: left atrium
LAA: left atrial appendage
LAD: left atrial dimension
MES: microembolic signal
PAF: paroxysmal atrial fibrillation
PeAF: Persistent atrial fibrillation
PV: pulmonary vein
PVI: pulmonary vein isolation
RF: radiofrequency
SCE: silent cerebral embolism
SCL: silent cerebral lesion
TCD: transcranial Doppler

## 1 INTRODUCTION

Atrial fibrillation (AF) is the most common arrhythmia in clinical practice and the prevalence of AF in the general population is increasing rapidly.^1^, ^2^ AF markedly increases the risk of ischemic strokes and death, and furthermore, it has been reported to be independently associated with cognitive dysfunction as well.^3^, ^4^, ^5^

Catheter ablation (CA) has become a standard therapeutic approach to AF.^2^ CA of AF can improve the quality of life of patients by maintaining sinus rhythm, and further, some observational studies have shown that AF ablation decreases the risk of ischemic strokes and death.^6^ Therefore, AF ablation has been increasingly performed worldwide. On the other hand, CA of AF imposes a low but relevant risk of thromboembolic complications of around 0.5%–1% including ischemic strokes, and has an additional risk of clinically silent cerebral embolisms (SCEs) of 10%–40%.^2^, ^7^, ^8^, ^9^ Although the mechanisms of the SCEs caused by CA of AF and its clinical impact are not fully understood, some studies have reported that SCEs could be a marker of procedure‐related clinical stroke risk, and cerebral microinfarctions due to those cerebral embolisms could contribute to cognitive dysfunction.^4^, ^10^, ^11^

Electrical pulmonary vein isolation (PVI) is the cornerstone of any AF ablative procedure not only for paroxysmal atrial fibrillation (PAF) but persistent atrial fibrillation (PeAF).^2^ Although the most widely adopted and established technique for AF ablation is radiofrequency (RF) ablation, the efficacy of CA in fact depends on the operator's experience, especially in cases of RF ablation.^12^ Recently, cryoballoon (CB; Arctic Front Advance, Medtronic) ablation has emerged to simplify the PVI and has also become a widely accepted strategy for CA of AF.^13^, ^14^, ^15^

Because there are fundamental differences in the two technologies of CB and RF ablation both in the energy form and energy delivery, the type and incidence rate of CA‐related complications differ between CB and RF ablation. There are few prospective data comparing the incidence of cerebral embolism after CA of AF between CB and RF ablation.

## 2 METHODS

### 2.1 Aim

The aim of the Embo‐Abl study is to compare the incidence of cerebral embolisms on 3 T diffusion‐weighted image (DWI) magnetic resonance imaging (MRI) after CA of AF between CB and RF ablation in patients with AF in a prospective, multicenter, open‐label, controlled, randomized, noninferiority fashion.

### 2.2 Primary hypothesis and study design

**Figure 1:**
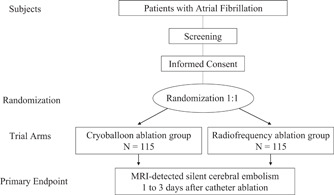
Flowchart of the study. MRI, magnetic resonance imaging.

The primary hypothesis of the Embo‐Abl study is that CB ablation is not inferior to RF ablation with respect to the incidence of cerebral embolisms after the CA of AF. The Embo‐Abl study is designed as a prospective, multicenter, open‐label, controlled, randomized, noninferiority clinical study comparing the incidence of periprocedural cerebral embolisms between CB and RF ablation of AF from April 28, 2021 to March 31, 2028 (Figure 1). The enrollment period will be from April 28, 2021 to March 31, 2025. The study cohort will include 230 patients with AF from a multicenter in Japan. The enrollment and study periods will be extended if the number of enrolled patients is not achieved during the period, and it will be shortened if the number of enrolled patients is achieved earlier.

### 2.3 Primary endpoint

The primary endpoint of the Embo‐Abl study will be the occurrence of MRI‐detected SCEs 1–3 days after CA.

### 2.4 Secondary endpoints

The key secondary endpoints are defined as follows:

*Safety analysis*:

1. Occurrence of silent cerebral lesions (SCLs) by brain MRI 1 to 3 days after CA of AF.
2. Occurrence of SCEs or SCLs by brain MRI 1 month after CA of AF.
3. Number of microembolic signals (MESs) during CA of AF.
4. Microbleeds detected by brain MRI 1 to 3 days and 1 month after CA of AF.
5. Complications following CA of AF.

*Efficacy analysis*:

1. The success rate of CA of AF during the acute phase.
2. Recurrence of atrial tachyarrhythmias (ATs) at 1 year after CA with a blanking period (BP) of 90 days after CA of AF.

### 2.5 Inclusion and exclusion criteria

#### 2.5.1 Inclusion criteria

Subjects must meet all the following criteria:

1. Diagnosed with AF with at least one episode documented (≥30 s) in accordance with the 2014 AHA/ACC/HRS guidelines.^2^
2. Planned RF or CB ablation of AF.
3. Age 20–85 years.
4. Capable of complying with the protocol and providing written informed consent.

#### 2.5.2 Exclusion criteria

Those who meet any of the following criteria are ineligible for the study:

1. History of AF ablation or cardiac surgery.
2. Left atrial dimension (LAD) > 55 mm (parasternal long‐axis view in transthoracic echocardiography).
3. Inability to undergo a brain MRI.
4. Women are currently or possibly pregnant.
5. Concomitant participation in another intervention study.
6. Patients whom doctors responsible for this study judge to be inappropriate.

### 2.6 Interventions

#### 2.6.1 Study procedures

Transesophageal echocardiography and/or contrast CT will be performed before the CA procedure in all patients to exclude the presence of any thrombi in the left atrial appendage (LAA) and left atrium (LA). All antiarrhythmic drugs except for amiodarone will be discontinued for at least five half‐lives. All patients will receive oral anticoagulants for at least 3 weeks before the CA procedure. Warfarin will be continued throughout the pre‐ and postprocedural period. Direct oral anticoagulants (DOACs) such as apixaban, rivaroxaban, dabigatran, or edoxaban will be continued throughout the pre‐ and postprocedural period, or the dose will be withheld on the day of the ablation procedure and will be resumed on the evening of the CA procedure or on the morning of the next day according to the hospital's standard strategy. The levels of the coagulation markers such as the prothrombin time, activated partial thromboplastin time, d‐dimer, thrombin‐antithrombin III complex, soluble fibrin monomer complex, and blood concentration of the DOACs will be measured before the CA procedure as preoperative samples, and 1–3 days after the procedure.

During the CA procedure, intravenous heparin will be introduced to maintain an activated clotting time (ACT) of 300–400 s during the procedure. The ACT will be monitored every 15 min. The esophageal temperature will be monitored with a temperature probe (SensiTherm Multi, Abbott; Esophastar, Japan Lifeline; Circa, Boston Scientific). A PVI will be mandatorily performed in both groups. Confirmation of LA to the pulmonary vein (PV) electrical connection and PVI will be conducted with a circular (Achieve, Medtronic, Lasso, Biosense Webster; Advisor, AFocus, Optima, Abbott; EP star Libero, Japan Lifeline), PentaRay (Biosense Webster), or HD grid (Abbott) catheter at a proximal site in the ostium in each vein before and after the ablation. Exit block from each PV is also confirmed after PVI by pacing from the circular, PentaRay, or HD grid catheter within the PV.

A cavotricuspid isthmus ablation will be performed in patients who have clinical or inducible cavotricuspid isthmus‐dependent atrial flutter. Other adjunctive ablation lesion sets targeting sites outside the PVs will be conducted when non‐PV AF triggers are detected and/or atrial tachycardias are induced.

#### 2.6.2 CB ablation

The detailed procedure has been described elsewhere.^16^ The fourth‐generation CB will be used in the CB ablation group. In brief, an 8.5‐F sheath (SL0, Abbott) will be exchanged for a 15‐F steerable sheath (FlexCath, Medtronic) after the transseptal puncture, and a 28‐mm CB will be introduced into the LA through the sheath. The CB will be inflated proximal to each PV and pushed gently to seal the antral aspect of the PV through a circular catheter (Achieve). Injection of contrast medium will be performed to confirm the relative position of the CB and PV antrum. A 180 s or time to isolation plus a 120‐s freeze cycle will be performed at each PV.^16^, ^17^ When the initial freezing fails to isolate the PV, the CB will be repositioned, and a second freezing cycle will be applied. When the CB ablation cannot achieve the PVI, additional focal ablation will be performed with a Freezor Max (Medtronic) or RF catheter.

#### 2.6.3 RF ablation

The detailed procedure has been described elsewhere.^18^ A three‐dimensional (3D) electroanatomic mapping will be performed with either the CARTO (Biosense Webster, Diamond Bat) or the NavX (Abbott) system. After the transseptal puncture, the 3D geometry and voltage map of the LA and PVs will be depicted by a circular (Lasso, Advisor, AFocus, Optima, EP star Libero), PentaRay, or HD grid catheter. The ablation of the ipsilateral superior and inferior PVs will be jointly performed under the navigation of the 3D mapping system. Electrical isolation of PV potentials and the bidirectional block will be confirmed with a multielectrode mapping catheter positioned sequentially in each PV.

RF ablation will be performed with an open irrigated tip catheter (Thermocool SmartTouch SF, Thermocool SmartTouch; Biosense Webster; TactiCath SE, Flexibility, Abbott). The RF ablation settings will be a power of 30‐50 W, targeting an ablation index of 450–550 for CARTO and a lesion index of 4.0–5.0 for NavX. The power and duration will usually be reduced to 20–25 W for 20 s on the LA posterior wall near the esophagus. Contact force data will be continuously monitored throughout the procedure to achieve at least 10 g (mean) with a vector perpendicular to the tissue and with an upper limit of 50 g.

#### 2.6.4 Postoperative brain MRI

Brain MRI will be acquired 1–3 days after the index ablation procedure using a 3 T scanner (MAGNETOM Prisma, Siemens Healthineers; MAGNETOM Vida, Siemens Healthineers; SIGNA Premier, GE Healthcare; Ingenia Elition, Philips Healthcare) with a six‐channel body array coil. The imaging protocol will consist of DWI, T2‐weighted axial fluid‐attenuated inversion recovery (FLAIR), and T2* (T2 star) sequences. The sequence parameters are described in the Supporting Information: Table.

For each DWI sequence, the apparent diffusion coefficient (ADC) map will be obtained to prevent overdetection of T2 shine‐through effects on the DWI. The slices and orientation of the DWI and FLAIR sequences will be matched to evaluate the correlation between the DWI and FLAIR images.

The number, localization, and size of the SCEs/SCLs will be analyzed.

According to the latest recommendations, SCEs/SCLs were defined as below:^7^

1. SCE = diffusion positive (DWI hyperintense) + ADC reduced and
2. SCL = diffusion positive (DWI hyperintense) + ADC reduced + FLAIR positive.

Patients with new embolic lesions on the postprocedural MRI will undergo a follow‐up MRI 1 month after CA to assess the lesion progression. Microbleeds will be identified and counted as nodular, strongly hypointense lesions on either the T2*‐weighted or susceptibility‐weighted imaging. All brain MRI images will be independently evaluated by certified radiologists (Yasutoshi Ohta and Tetsuya Fukuda) blinded to the patient characteristics and ablation technology used. If the SCEs and/or SCLs are detected on brain MRI, a neurologist or cardiologist who has been trained in neurology will assess the neurological findings and determine whether they are symptomatic or asymptomatic.

#### 2.6.5 Transcarotid echography

We will use a probe and ultrasound system (FUtuRe Ultrasound HArmonic Thromboembolus Analyzer, FURUHATA, HDK‐BM001; Hashimoto Electronic Industry Co., Ltd.) that will attach to the neck and detect MESs at the carotid artery.^19^, ^20^ The probe is rectangular parallelepiped, 22 mm in depth,31 mm in width, and 12 mm in height. The probe acts as an acoustic prism between the surface of the probe and neck skin by attaching a soft silicone wedge and transmits the ultrasound beam with an incidence angle of around 30 degrees to the human body. The properties of the FURUHATA are the same as those of transcranial Doppler (TCD) (center frequency 2.0 MHz, pulse repetition frequency 5.0 kHz, and sample volume 7 mm). The system includes the machine body, operation and display screen, isolation transformer, and probe (HDK‐MB001; Hashimoto Electronic Industry Co., Ltd.). The sample volume is user‐configurable between 3.5 and 14 mm, and the insonation depth will be 10.5–65 mm. The Doppler waveforms of all the saved embolic signals will be recorded onto the hard drive of the computer. All signals will be reviewed by observers (Akihisa Narai and Tomohiro Nakase) experienced in MES detection and will be blinded to the patient characteristics and ablation technology used.

The MES counts will be collected and evaluated separately during the different steps of the CA procedure as follows: (1) catheter manipulation and sheath flush in the right atrium; (2) transseptal puncture; (3) introducing the sheaths (SL0, Agilis, Vizigo, and/or FlexCath) into the LA; (4) introducing the CB/Achieve into the LA for CB ablation; (5) CB inflation and PV occlusion for CB ablation; (6) contrast injection to optimize the CB position for CB ablation; (7) freezing phase for CB ablation; (8) thawing phase for CB ablation; (9) CB deflation for CB ablation, Steps (4)–(9) will be repeated until achieving isolation of all four PVs; (10) obtain a 3D geometry depiction of the LA and PVs for RF ablation; (11) RF energy applications for the right PVI for RF ablation; (12) RF energy applications for the left PVI for RF ablation; and (13) induction and ablation of non‐PV triggers using isoproterenol. If a contrast injection of the entire LA is performed, MES measurements will be performed at that time as well. MESs will be divided into thrombotic debris (clots and char) as high‐intensity transient signals or microbubbles.

### 2.7 Follow‐up

Personal study visits will be scheduled for 1 month, and then every 1–3 months following the CA procedure with clinic visits. Study visits will include a medical history, physical examination, 12‐lead electrocardiograms (ECGs), and 24‐h Holter recordings (3 and 12 months after the procedure). When patients report any symptoms, one‐channel ambulatory ECGs (HCG‐801; OMRON Healthcare) will be obtained. A BP of 90 days following the index procedure is defined. Recurrence of ATs is defined as any documented ATs lasting ≥30 s occurring outside the BP. In the case of recurrences outside the BP, it will be recommended to perform a second CA.

Anticoagulation will be continued for at least 3 months. The discontinuation of the anticoagulant will be decided based on the CHADS_2_ and CHA_2_DS_2_‐VASc score unless recurrence of ATs will be observed. Adverse events (AE) including complications will be collected during the study periods (Table 2). An AE is defined as any untoward medical occurrence in a subject in this study, regardless of whether there is a causal relationship of the AE with the ablation procedure.

### 2.8 Sample size and power

The targeted sample size is 230 (115 in each group). The sample size was calculated based on the primary hypothesis. The rate of MRI‐detected SCEs after CA of AF has been reported to be comparable between CB and RF ablation, ranging from 10% to 30% in the observational studies.^7^ Based on those data, a rate of MRI‐detected SCEs of 20% in both groups is assumed. The noninferiority hypothesis will be evaluated with a noninferiority margin of 15%. The sample size is calculated as 112 patients per group with a power of 80% based on a significance level of 5%. To cope with a potential loss‐to‐follow‐up, a minimum of 230 patients (115 patients per group) will be enrolled in this study.

When the primary endpoint is observed in half of the total sample size (116 patients), early stopping of the study for futility will be considered based on the conditional power at an interim analysis, which will be reviewed by the Data and Safety Monitoring Board (DSMB). A simulation suggested a very limited power loss with a potential early stopping for futility, and the targeted power is expected to be achieved with this sample size.

### 2.9 Randomization and allocation factor

The patients will be registered and randomly assigned to either the CB or RF ablation group in a 1:1 ratio using a UMIN INDICE cloud system. The treatment allocation will be based on a covariate‐adaptive randomization (minimization) scheme including the age (<70 vs. ≥70 years), AF type (PAF vs. PeAF), and LAD (<50 vs. ≥50 mm), as covariates.^21^

### 2.10 Data quality control and management

The principal investigator will authorize access to the electronic Case Report Form (CRF) system for investigators. The principal investigator will take full responsibility for the accuracy and reliability of all the data entered in the CRFs. The principal investigator and other investigators must not disclose the information contained in the CRFs to third parties. Only investigators can access data.

### 2.11 Statistical analysis

For the primary endpoint, safety analyses will be performed based on the intention‐to‐treat principle. A per‐protocol basis analysis will also be performed as a supplemental analysis. When noninferiority is demonstrated on the primary endpoint, the superiority hypothesis will also be evaluated. An asymptotic method based on the normal distribution will be used to compare the rate of SCEs by brain MRI 1–3 days after CA of AF.

Other categorical variables will be presented as absolute and relative frequencies and compared using a chi‐square test or Fisher's exact test. Continuous variables will be presented as the mean ± standard deviation for normally distributed data or median with the interquartile range (25th–75th percentiles) for skewed data and analyzed with the Student's *t*‐test or Wilcoxon rank‐sum test, as appropriate. If RF catheters are used in the CB ablation group for touch‐ups, we plan to carry out analyses that include both patients with touch‐ups and that excludes those without touch‐ups, respectively.

A *p* < .05 will be considered statistically significant. Logistic regression analyses and multivariable Cox regression analyses will be performed to investigate the predictors associated with clinical outcomes. Statistical analysis will be performed using up‐to‐date versions of the Stata software (StataCorp). The patient demographic data and outcomes of the CA in each group will be collected descriptively as presented in Tables 1 and 2. A detailed plan for the interim analysis and final analysis will be prespecified in the statistical analysis plan, which will be prepared and finalized before the database lock.

**Table 1:** Patient characteristics at baseline

Age, years, *n* (%)
Female sex, *n* (%)
Height, cm
Weight, kg
Body mass index
AF type
Blood pressure, mmHg
Heart rate, /min
Congestive heart failure, n (%)
Hypertension, *n* (%)
Diabetes mellitus, *n* (%)
Stroke and/or transient ischemic attack, *n* (%)
Vascular disease, *n* (%)
Structural heart disease, *n* (%)
Coronary artery disease
Valvular heat disease
Dilated cardiomyopathy
Hypertrophic cardiomyopathy
Others
Post‐open heart surgery
NYHA class
Bleeding history or predisposition
Labile INR, *n* (%)
Concomitant aspiring or nonsteroidal anti‐inflammatory drugs
Excess alcohol use
CHADS_2_ score
CHA_2_DS_2_‐VASc score
HAS‐BLED score
Transthoracic echocardiographic data
LV ejection fraction, %
LV end‐diastolic diameter
LV end‐systolic diameter
LA dimension, mm (%)
LA volume, ml
Transmitral flow
Mitral valve regurgitation
Tricuspid valve regurgitation
Transesophageal echocardiographic data
LA appendage inflow velocity
LA appendage outflow velocity
Spontaneous echo contrast
Cardiac implantable electronic device, n (%)
Pacemaker, *n* (%)
ICD, *n* (%)
CRT‐P, *n* (%)
CRT‐D, *n* (%)
Type and dose of anticoagulant
History of antiarrhythmic drug use, *n* (%)
Disopyramide, *n* (%)
Cibenzoline, *n* (%)
Aprindine, *n* (%)
Pilsicainide, *n* (%)
Flecainide, *n* (%)
Propafenone, *n* (%)
Bepridil, *n* (%)
Sotalol, *n* (%)
Amiodarone, *n* (%)
Verapamil, *n* (%)
Beta‐blocker, *n* (%)
Digitalis, *n* (%)
Others, *n* (%)
Laboratory data

Abbreviations: AF, atrial fibrillation; CRT‐D, cardiac resynchronization therapy defibrillator; CRT‐P, cardiac resynchronization therapy pacemaker; ICD, implantable cardioverter defibrillator; INR, international normalized ratio; LA, left atrium; LV, left ventricle; NYHA, New York Heart Association.

**Table 2:** The CA procedure and complications

Procedure time (groin puncture to catheter extraction), min
LA dwell time, min
Fluoroscopic time, min
General anesthesia, *n* (%)
For CB ablation
Total freeze cycles, *n*
Total freeze time, s
The need for touch‐up ablation, *n*
Reinsertion of withdrawn CB
Mapping of LA before ablation
Mapping of LA after ablation
For RF ablation
Type of ablation catheter
Total RF application time, s
Power, W
Duration at each application site, s
Ablation index for CARTO, lesion index for NavX
Other adjunctive ablation, *n* (%)
Use of 3D mapping system, *n* (%)
Low voltage zone ≥10% of LA surface
Electrical cardioversion during CA procedure, *n* (%)
Number of cardioversion during CA procedure, *n* (%)
Initial heparin dose, U
Time required to reach target ACT of >300, min
Mean ACT value during CA procedure
Total heparin dose, U
Use of protamine at the end of CA procedure, n (%)
Transcarotid echography during CA procedure
Microembolic signals, *n*
High‐intensity transient signals (microparticles), n
Microbubbles, *n*
Brain MRI
Silent cerebral events, *n* (%)
Location and number of silent cerebral events
Silent cerebral lesions, *n* (%)
Location and number of silent cerebral lesions
Microbleeds, *n* (%)
Location and number of microbleeds
Complications, *n* (%)
Pericardial effusion requiring drainage
Pericardial effusion not requiring drainage
Transient ischemic attack
Cerebral infarction
Other thromboembolisms
Transient phrenic nerve paralysis
Prolonged phrenic nerve paralysis
Severe pulmonary vein stenosis
Hematoma at the puncture site
Pseudoaneurysm at the puncture site
Gastric hypomotility
Transient ST elevation (transient coronary air embolism)
Others
Death
Discharge prescription, *n* (%)
Oral anticoagulant
Vitamin‐K antagonist
Direct oral anticoagulant
Antiarrhythmic drugs
Disopyramide
Cibenzoline
Aprindine
Pilsicainide
Flecainide
Propafenone
Bepridil
Sotalol
Amiodarone
Verapamil
Beta‐blocker
Digitalis
Others
Angiotensin‐converting enzyme inhibitor
Angiotensin II receptor blocker
Angiotensin receptor‐neprilysin inhibitor
Mineralocorticoid receptor antagonists
Ivabradine
Loop diuretic
Statin

Abbreviations: 3D, three‐dimensional; ACT, activated clotting time; CA, catheter ablation; CB, cryoballoon; LA, left atrium; MRI, magnetic resonance imaging; RF, radiofrequency.

### 2.12 Study organization

The research group consists of investigators at a multicenter in Japan and an independent data monitoring committee. The Certified Review Board (CRB) and DSMB will regularly monitor the recruitment and conduct of the study, data quality, timeliness, distribution of the therapies within the trial groups, and serious AE related to the CA procedure.

### 2.13 Ethics

The study is registered in the Japan Registry of Clinical Trials (jRCT) identifier (jRCT1052210013). The study is being conducted in accordance with the Declaration of Helsinki and the Ethical Guidelines for Clinical Studies issued by the Ministry of Health, Labour and Welfare, Japan. This study received approval from the CRB of the Osaka City University Hospital, Japan (OCU007E, April 6, 2021). A CRB‐approved informed consent form (written in accordance with the applicable laws for clinical research) will be obtained from every patient before study enrollment.

## 3 RESULTS

The results of this study are currently under investigation.

## 4 DISCUSSION

The mechanisms of cerebral embolisms caused by the CA of AF are still under debate. Different technology‐, procedure‐, and patient‐related factors are considered to play roles in the multifactorial genesis of cerebral embolisms caused by CA of AF.^22^, ^23^ The potential embolic sources due to CA of AF are considered to be the development of thrombotic debris (clots and char) and air microbubbles, which are introduced or produced during CA procedures.^7^, ^24^ Haines et al.^24^ reported that both thrombotic debris and air microbubbles produced SCLs detected by MRI in a canine model.

Regarding the development of thrombotic debris (clots and char) during CA procedures, endothelial injury and tissue overheating, systemic activation of platelets and of the coagulation system, and debris introduced from within the transseptal sheath or catheter, may cause cerebral embolism.^22^, ^25^ Regarding the development of microbubbles during CA procedures, it has been reported that microbubbles can be introduced into the LA by a variety of mechanisms associated with introducing, withdrawing, and exchanging catheters within the transseptal sheath.^26^, ^27^

Some procedure‐ and patient‐related factors have been also reported as possible predictors of cerebral embolisms caused by CA of AF.^8^, ^27^, ^28^, ^29^ As procedure‐related factors, cardioversion during CA, lower ACT levels during CA, heparin application after the transseptal puncture, higher blood pressures during CA, type of oral anticoagulant, interrupted anticoagulation before CA, use of catheters with complex geometries, the LA dwell time, negative pressure in the LA caused by general anesthesia, use of RF energy during the transseptal puncture, additional linear LA lesions, and prolonged procedure time have been reported as risk factors of CA related embolisms, however, these relationships are controversial as well.^8^, ^27^, ^28^ As patient‐related factors, a history of a stroke or transient ischemic attack, higher CHADS_2_ and CHA_2_DS_2_‐VASc scores, male gender, hypertension, congestive heart failure, diabetes, coronary artery disease, PeAF, larger LA volume, spontaneous LAA echo contrast in preablation transesophageal echo, and LA scarring have also been reported as risk factors of CA related embolisms.^7^, ^30^, ^31^

The pathogenesis of cerebral embolisms caused by the CA of AF is incompletely understood. Therefore, periprocedural monitoring is expected to be a useful approach to determine which part of the CA procedure is associated with MESs, although all MESs does not result in cerebral micro‐embolisms because of collateral circulation via the circle of Willis in the cerebral vasculature.^24^ TCD is an available method for real‐time monitoring of the embolic traffic in the brain. Miyazaki et al.^32^ used TCD throughout all CB ablation procedures in their observational study. They reported that a considerable number of MESs were recorded during a variety of steps throughout the procedure, and the total MES count/procedures reached over 500 per CA procedure.^32^ In this way, TCD is useful for real‐time monitoring during CA, however, monitoring throughout entire relatively long CA procedures such as RF ablation is hard to perform because TCD needs manual adjustment of the probe throughout the procedure. In this study, we will use a novel probe that will attach to the neck easily and be stable throughout the procedure, which will enable real‐time monitoring of the embolic traffic in both the CB and RF ablation groups. In addition to monitoring for MESs throughout the procedure and assessing how the CA procedure is associated with promoting MESs, we will assess the relationship between MESs during the CA procedure and cerebral embolisms detected by DWI‐MRI after the CA.^33^

Both CB and RF ablation is used worldwide, however, different technologies used for CA of AF have shown different characteristics of the complications.^34^ We will compare the incidence of cerebral embolisms caused by CA of AF between CB and RF ablation with the Embo‐Abl study.

## 5 LIMITATIONS

We will not acquire brain MRI before CA in this study, and therefore, it is possible that postprocedural SCEs/SCLs may occur preprocedural. However, we think that is highly unlikely because acute cerebral lesions will be defined on 3 T DWI, which is positive only a few weeks after a cerebral embolism, and actually, acute cerebral lesions were found in none of the previous studies that assessed preprocedural MRI in patients who underwent CA of AF.^35^

## 6 CONCLUSION

The Embo‐Abl study will be the first to compare the incidence of periprocedural cerebral embolisms caused by CA of AF between CB and RF ablation in a prospective, multicenter, randomized, controlled fashion.

## CONFLICT OF INTEREST

Satoshi Nagase belongs to a donation course at Medtronic. Takeshi Aiba belonged to a donation course at Medtronic. Kengo Kusano report lecture/consultant/advisory honoraria from Medtronic.

## Supporting information

Supporting Information.

## Data Availability

The data that support the findings of this study are available from the corresponding author upon reasonable request.
