# Supplementary material for: A multicenter prospective randomized study comparing the incidence of periprocedural cerebral embolisms caused by catheter ablation of atrial fibrillation between cryoballoon and radiofrequency ablation (Embo‐Abl study): Study design
Source: Clin Cardiol. 2022 Nov 22;46(2):214–22. doi: 10.1002/clc.23949 (PMC9933100; doi:10.1002/clc.23949)
Supplement: Supplementary file 1 — Supporting Information. [file CLC-46-214-s001.docx]

Diffusion weighted image

|  | MAGNATOM Prisma | MAGNETOM Vida | Ingenia Elition | SIGNA Premier |
| --- | --- | --- | --- | --- |
| TR/TE (ms) | 5000/80 | 5000/82 | 4809/61 | 5000/80 |
| Flip angle(°) | 90 | 90 | 90 | 90 |
| Bandwidth (Hz/Px) | 1840 | 1359 | 1994 | 1953 |
| Acquisition matrix | 160x130 | 160x130 | 152x132 | 160x130 |
| FOV (mm) | 230x261 | 230x261 | 271x230 | 272x230 |
| Slice thickness | 4 | 4 | 4 | 4 |
| NEX | 2 | 1 | 1 | 1 |
| b-value | 0, 1000 | 0, 1000 | 0, 1000 | 0, 1000 |

TE = echo time, TR = repetition time, FOV = field of view NEX = number of excitations

Fluid attenuated inversion recovery (FLAIR) image

|  | MAGNETOM Prisma | MAGNETOM Vida | Ingenia Elition | SIGNA Premier |
| --- | --- | --- | --- | --- |
| TR/TE (ms) | 12000/116 | 12000/119 | 10000/130 | 12000/119 |
| Inversion time (ms) | 2800 | 2800 | 2700 | 2800 |
| Flip angle (°) | 150 | 150 | 90 | 150 |
| Bandwidth (Hz/Px) | 400 | 401 | 218 | 195 |
| Acquisition matrix | 320x189 | 320x189 | 304x219 | 320x189 |
| FOV (mm) | 203x230 | 203x230 | 272x230 | 272x230 |
| Slice thickness (mm) | 4 | 4 | 4 | 4 |
| NEX | 1 | 1 | 1 | 1 |

T2* weighted image

|  | MAGNETOM Prisma | MAGNETOM Vida | Ingenia Elition | SIGNA Premier |
| --- | --- | --- | --- | --- |
| TR/TE (ms) | 550/12 | 550/12 | 400/11.51 | 550/20 |
| Flip angle(°) | 20 | 20 | 20 | 20 |
| Bandwidth (Hz/Px) | 230 | 230 | 217 | 139 |
| Acquisition matrix | 320x260 | 320x160 | 288x230 | 320x260 |
| FOV (mm) | 203x230 | 203x230 | 272x230 | 272x230 |
| Slice thickness(mm) | 4 | 4 | 4 | 4 |
| NEX | 1 | 1 | 1 | 1 |

|  | MAGNETOM Prisma | MAGNETOM Vida | Ingenia Elition 3.0T | SIGNA Premier |
| --- | --- | --- | --- | --- |
| Coil | Head/Neck 64 | Head neck 64ch | dS Head neck 32ch coil | 48ch Head coil 3.0T |
